# A multimodal imaging workflow for monitoring CAR T cell therapy against solid tumor from whole-body to single-cell level

**DOI:** 10.7150/thno.68966

**Published:** 2022-06-13

**Authors:** Rita Pfeifer, Janina Henze, Katharina Wittich, Andre Gosselink, Ali Kinkhabwala, Felix Gremse, Cathrin Bleilevens, Kevin Bigott, Melanie Jungblut, Olaf Hardt, Frauke Alves, Wa'el Al Rawashdeh

**Affiliations:** 1 Miltenyi Biotec B.V. & Co. KG, R&D Reagents, Bergisch Gladbach, North Rhine-Westphalia, Germany.; 2 University Medical Center Göttingen, Translational Molecular Imaging, Institute for Diagnostic and Interventional Radiology & Clinic for Haematology and Medical Oncology, Göttingen, Lower Saxony, Germany.; 3 Institute of Medical Statistics and Computational Biology, University of Cologne, Cologne, North Rhine-Westphalia, Germany.; 4 Gremse-IT GmbH, Aachen, North Rhine-Westphalia, Germany.; 5 Max-Planck-Institute for Multidisciplinary Science, Translational Molecular Imaging, Göttingen, Lower Saxony, Germany.; 6 Ossium Health Inc, Indianapolis, Indiana, United States of America.

**Keywords:** CAR T Cells, Cell Tracking, Optical tomography, 3D µCT/BLT, Light-Sheet Fluorescence microscopy

## Abstract

CAR T cell research in solid tumors often lacks spatiotemporal information and therefore, there is a need for a molecular tomography to facilitate high-throughput preclinical monitoring of CAR T cells. Furthermore, a gap exists between macro- and microlevel imaging data to better assess intratumor infiltration of therapeutic cells. We addressed this challenge by combining 3D µComputer tomography bioluminescence tomography (µCT/BLT), light-sheet fluorescence microscopy (LSFM) and cyclic immunofluorescence (IF) staining.

**Methods:** NSG mice with subcutaneous AsPC1 xenograft tumors were treated with EGFR CAR T cell (± IL-2) or control BDCA-2 CAR T cell (± IL-2) (n = 7 each). Therapeutic T cells were genetically modified to co-express the CAR of interest and the luciferase CBR2opt. IL-2 was administered s.c. under the xenograft tumor on days 1, 3, 5 and 7 post-therapy-initiation at a dose of 25,000 IU/mouse. CAR T cell distribution was measured in 2D BLI and 3D µCT/BLT every 3-4 days. On day 6, 4 tumors were excised for cyclic IF where tumor sections were stained with a panel of 25 antibodies. On day 6 and 13, 8 tumors were excised from rhodamine lectin-preinjected mice, permeabilized, stained for CD3 and imaged by LSFM.

**Results:** 3D µCT/BLT revealed that CAR T cells pharmacokinetics is affected by antigen recognition, where CAR T cell tumor accumulation based on target-dependent infiltration was significantly increased in comparison to target-independent infiltration, and spleen accumulation was delayed. LSFM supported these findings and revealed higher T cell accumulation in target-positive groups at day 6, which also infiltrated the tumor deeper. Interestingly, LSFM showed that most CAR T cells accumulate at the tumor periphery and around vessels. Surprisingly, LSFM and cyclic IF revealed that local IL-2 application resulted in early-phase increased proliferation, but long-term overstimulation of CAR T cells, which halted the early added therapeutic effect.

**Conclusion:** Overall, we demonstrated that 3D µCT/BLT is a valuable non-isotope-based technology for whole-body cell therapy monitoring and investigating CAR T cell pharmacokinetics. We also presented combining LSFM and MICS for *ex vivo* 3D- and 2D-microscopy tissue analysis to assess intratumoral therapeutic cell distribution and status.

## Introduction

Over the last decade, CAR T cell therapy has emerged as a mainstay in the treatment of advanced B cell-derived malignancies prompting a translation of this treatment option to the solid tumor setting. Despite significant effort, however, a corresponding success in the solid cancer field has failed to materialize. Several factors have contributed to this failure with the lack of truly tumor-specific antigens, inefficient T cell trafficking to the tumor site and a hostile tumor microenvironment (TME) being the predominant obstacles 1-10. To overcome these barriers a plethora of approaches have been developed, but the clinical experience has remained disappointing 11-15. The chief obstacle has been a lack of knowledge surrounding the *in vivo* characteristics of the therapeutic cells and as an inevitable consequence, many lessons have been learned “the hard way” in the clinical setting. For example, using highly potent CAR T cells to overcome the immunosuppressive TME led to severe on target/off tumor toxicities and uncontrolled T cell proliferation with partially lethal outcomes 3, 16. Neurotoxicities associated with CD19 CAR T cell therapies have long been believed to be the result of cytokine release syndrome (CRS)-induced disruption of the blood-brain-barrier (BBB) leading to an influx of CAR T cells into the brain 17-19. However, recent data suggests the presence of CD19-positive mural cells in the BBB that are actively attacked by the therapeutic cells in this way revealing unexpected on target/off tumor toxicities 20. A relatively new phenomenon that is getting unveiled is pseudoprogression. It is a radiologically observed increase in tumor volume of lymphoma patients following CAR T cell administration which is due to CAR T cell proliferation and other immune cell infiltration upon tumor recognition 21-25. Although remission eventually follows, in clinical practice, enlargement of a lesion site is interpreted as progressive disease and treatment change is usually recommended. Thus, pseudoprogression bears the risk of clinical misinterpretation and premature cessation of effective therapies.

In light of these experiences, the need is growing for preclinical evaluation systems that allow a profound analysis of the therapeutic cells' biodistribution, their long-term viability as well as their biological state concerning their activation and differentiation status in different organs. Imaging platforms with spatiotemporal information can provide these insights and help to accelerate the advancement of the most promising therapies to the clinic. Due to the clinical relevance of positron emission tomography (PET) and single-photon emission computed tomography (SPECT), several groups have focused on investigating CAR T cell tracking strategies using radionuclide-based imaging. Introducing reporter genes that are passed with cell division, the investigators followed the dynamics of CAR T cell behavior until late time points of therapy and revealed events that are understudied as of yet or occur in a time-displaced fashion 26-31. Specifically, Volpe and colleagues demonstrated differential retention rates of CAR T cells in distinct triple negative breast cancer (TNBC) entities resulting in divergent therapeutic efficacy 32 while Park and colleagues proved longitudinal imaging applicable to identify CAR therapies with different therapeutic indexes including the assessment of on target/off tumor toxicities 26.

Despite the enormous potential of PET/SPECT, they face major limitations such as ionizing radiation and high system/scan costs preventing preclinical screenings in higher throughput 33. Thus, optical imaging is establishing itself as a facile and inexpensive alternative that allows the analysis of multiple animals simultaneously. In this regard, optical luciferase-based reporter gene systems were successfully applied to characterize CAR T cell responses even at low T cell numbers and for a prolonged period of time 34, 35. Yet, a drawback of these studies was poor spatial resolution that was restricted to the 2-dimensional (2D) space and complicated precise delineation of the therapeutic cells' localization. Besides, the qualitative nature of optical imaging disables the tracking of cells at a single cell and sub-organ level. However, these aspects become increasingly important for the optimization of cellular therapies, as it has been shown that spatial organization of therapeutic cells within tumor entities impacts the clinical outcome 36, 37. In particular, three main spatial tumor phenotypes have been recognized for their clinical relevance: inflamed (presence of intratumoral lymphocytes), excluded (lymphocytes are restricted to the invasive margin) and ignored (lack of intratumoral lymphocytes). Moreover, emerging data suggests that besides frequency and spatiality, also the functional phenotype of the intratumoral immune cells has a prognostic value and should thus be taken into consideration 36, 38. Specifically, PD-1 in combination with LAG3 and/or TIM3 expression was shown to differentiate between activated and exhausted intratumoral T cells and define the choice of therapy 39-43.

Collectively, the aforementioned findings highlight that multiscale and multimodal imaging approaches are necessary for a comprehensive evaluation of CAR T cell therapies. Therefore, in this study, we aimed to establish a multimodal imaging workflow that allows spatiotemporal analysis of CAR T cell therapy from the whole-body to the single-cell level. For this, we combined longitudinal 3D µCT/BLT for whole-mouse imaging with subsequent endpoint LSFM and MICS analysis for single cell monitoring and phenotyping. BLT is superior to BLI as it provides anatomical resolution in 3D and allows for signal quantitation at the organ level 44, 45. LSFM was integrated for in-depth characterization of CAR T cell infiltration in the tumors and MICS served to deep-phenotype the intratumoral T cells 46, 47. The workflow was applied to investigate the behavior of therapeutic EGFR CAR T cells in comparison to control BDCA-2 CAR T cells in a subcutaneous pancreatic cancer xenograft mouse model. Moreover, the impact of additional IL-2 supply at the tumor site for T cell stimulation was evaluated. The multifactorial insights gained on the *in vivo* behavior of the CAR T cells prove the imaging platform advantageous to obtain a holistic and detailed understanding of the interaction of therapeutic cells in their *in vivo* environment as well as their anti-tumor function.

## Material and Methods

### Transgene Construction

**Figure 1:**
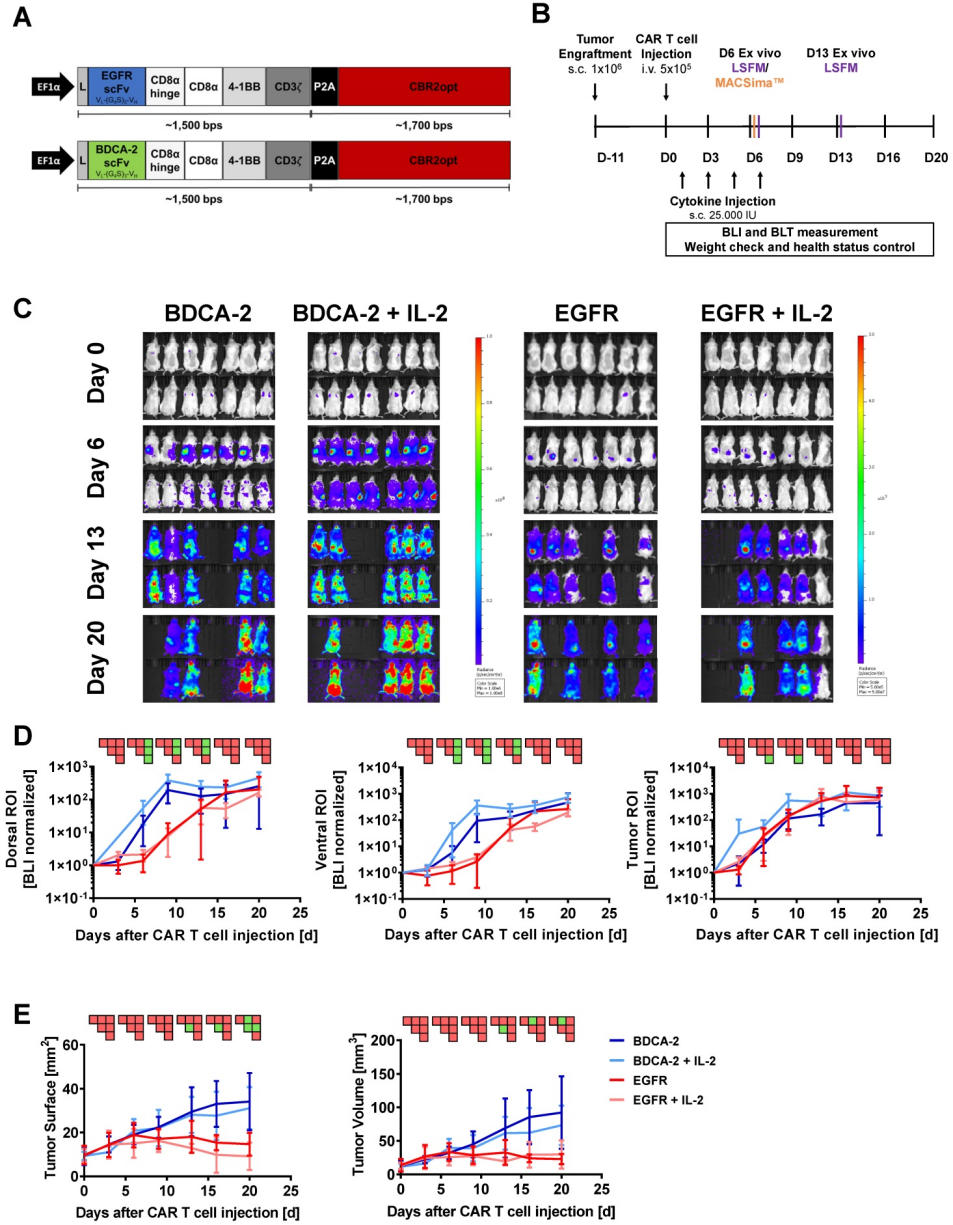
** 2D BLI fails to correlate with therapeutic efficacy of EGFR CAR T cells within the tumor.** Although distinct intratumoral signals were seen in the EGFR CAR T cells treated groups, 2D BLI tumor ROI analysis failed to measure a difference in BLI signal between EGFR treatment and control groups. **(A)** Structure of EGFR and BDCA-2 CARs. **(B)** Schematic representation of the *in vivo* study timeline in the pancreatic xenograft tumor model. Mice were randomized in four groups (n = 7/each) receiving either EGFR or BDCA-2 CAR T cells, with or without IL-2 s.c. application. On day 6 mice (n = 1/group) were sacrificed and tumors were excised for MACSima™ analysis. On days 6 and 13 mice (n = 1/group/day) were sacrificed and tumors were excised for LSFM analysis. **(C)** *In vivo* proliferation of CBR2opt-transduced CAR T cells was analyzed by 2D BLI (p/sec/cm^2^/sr). Scale factor: BDCA-2 groups: min: 1∙10^6^, max: 1∙10^8^, EGFR groups: min: 5∙10^5^, max: 5∙10^7^. **(D)** Quantification of signal amplification in the dorsal, ventral and tumor ROIs over time. **(E)** Tumor burden change, measured by surface area using a 2D Caliper. For sake of later comparison with 3D µCT measurements, tumor volumes were calculated with the formula V = ½ (Length × Width^2^). PSM p < 0.05 (green).

Bicistronic transgenes were designed to coexpress the CAR of interest and the mutant version of click beetle red luciferase CBR2opt in T cells 48. The CARs were composed of a second-generation architecture that contained the CD8α hinge and transmembrane domain in conjunction with the 4-1BB and CD3ζ signaling domains. The EGFR-specific scFv was derived from the clinical antibody Cetuximab 49, while the BDCA-2 specific scFv was obtained from the humanized AC144 clone 50. V_L_ and V_H_ regions of the antigen-binding domains were connected via a (G_4_S)_3_-linker. A P2A sequence was engineered between CAR and luciferase to ensure efficient separation upon transgene translation (Figure 1A). Expression of the transgene was driven by the EF1α promoter. CBR2opt sequence was obtained from Addgene and gene fragments for cloning were obtained commercially (ATUM).

### Lentiviral Vector Production

Lentiviral vector production was performed as previously described 51. In brief, HEK293T cells at a confluency of 70-90% were transfected with DNA:MACSfectin™ (Miltenyi Biotec) complexes using a total amount of 50µ DNA per T175 flask. HEK293T cells were then incubated overnight and sodium butyrate was added at a final concentration of 10 mM. Lentiviral vector containing supernatant was collected 48-60 h after transfection and following a filtration through 0.45 μm-pore-size PVDF filters subjected to centrifugation at 4 °C and 4,000×g for 24 h. Air-dried pellets containing lentiviral particles were resuspended at a 200-fold concentration with 4 °C cold PBS, aliquoted and stored at -80 °C.

### CAR T Cell Generation

Buffy coats from consenting healthy anonymous donors were obtained from the German Red Cross Dortmund as registered and approved by the Ethics Committee of the German Red Cross. All study-related procedures have been performed according to the Declaration of Helsinki and to the relevant ethical guidelines.

CAR T cells were generated according to previously applied protocols 46. In brief, peripheral blood mononuclear cells (PBMCs) were isolated from *buffy coats* by density gradient centrifugation and subsequent T cells enrichment was performed using the human pan T Cell Isolation Kit (Miltenyi Biotec) according to the manufacturer's instructions. Isolated T cells were cultivated in TexMACS^TM^ Medium (Miltenyi Biotec) supplemented with 200 IU/ml of recombinant human IL-2 IS, research grade (Miltenyi Biotec) and activated with TransAct^TM^, human (Miltenyi Biotec). Twenty-four hours after activation, lentiviral vector was added for transduction. T Cell TransAct^TM^ and excess viral vector were removed 3 days post activation and replaced with fresh TexMACS^TM^ Medium, supplemented with 200 IU/mL IL-2, for further cultivation of T cells. Cell splitting and feeding occurred in a 2-3 days interval. After 12 days of expansion, T cells were subjected to experimental studies.

### Flow Cytometry

Cellular composition of purified T cells was analyzed with hCD3, hCD4 and hCD8 REAfinity recombinant antibodies (Miltenyi Biotec) as previously reported 46. CAR expression was detected by a sequential staining protocol. Transduction efficiency of anti-EGFR CAR T cells was determined with a recombinant EGFR protein including a His-Tag (AcroBiosystems), followed by a monoclonal anti-His antibody (Miltenyi Biotec). BDCA-2 CAR T cells were identified by a biotin-tagged BDCA-2 CAR Detection Reagent (Miltenyi Biotec) followed by an anti-Biotin antibody (Miltenyi Biotec). For stainings, samples were incubated with the respective primary antibody at manufacturer recommended concentrations for 10 min at 4 °C. Samples were washed 2× and incubated with the respective secondary antibodies at recommended concentrations for 10 min at 4 °C. Stained cells were acquired on a MACSQuant Analyzer 8 (Miltenyi Biotec) and analyzed using the MACSQuantify^TM^ Software 2.13v.

### *In vivo* analysis

#### Tumor mouse model

All experiments were performed according to guidelines and regulations and were approved by the Governmental Review Committee on Animal Care in North Rhine Westphalia (NRW), Germany (Landesamt für Natur, Umwelt and Verbraucherschutz NRW, Approval number 84-02.04.2017.A021). NOD SCID gamma (NSG; NOD.Cg-PrkdcscidIl2rgtm1Wjl/SzJ) mice (Jackson Laboratory, provided by Charles River) were injected subcutaneously (s.c.) with 1∙10^6^ wildtype AsPC1 cells in 100 µL PBS in the right flank for tumor establishment 52. Tumor growth was tracked by 2D caliper measurements over time. When tumors reached a size of > 25 mm^2^, mice were randomized and freshly prepared CAR T cells were injected in 100 µL PBS into the tail vein. In the cytokine-treated cohorts, IL-2 was administered s.c. under the xenograft tumor on day 1, 3, 5 and 7 post therapy initiation at a dose of 25,000 IU/mouse. Throughout the experiment, the well-being of mice was monitored and scored according to relevant animal care guidelines. Animals were euthanized according to guidelines after 20 days or upon reaching the endpoint (paralysis, stress score of > 20, weight loss of > 19%, or endpoint of the experiment) and tumors were excised for further *ex vivo* analysis.

### 2D and 3D bioluminescence imaging

CAR T cell biodistribution was analyzed in 2D and 3D on day 0, 3, 6, 9, 13, 16 and 20 after intraperitoneal (i.p.) injection of 100 μL of 30 mg/mL D-Luciferin Potassium Salt LUCK (GoldBio) in isoflurane-anesthetized mice as described before 51. 2D measurements were performed using the *in vivo* imaging system (IVIS) Lumina III (PerkinElmer) with open filters 8 min after substrate injection. 2D data were analyzed and quantified with living image software 4.7.3v (PerkinElmer). For comparison of the different treatment groups, total flux of dorsal, ventral and tumor region of interest (ROI) were normalized to the value measured on day 0.

For 3D measurements, isoflurane-anesthetized mice were transferred to a three-mouse-bed of the optical imaging (OI) module, which was connected to the µCT module (MILabs). 3D BLT signal was acquired according to the manufacturer's protocol with emission filters at 586, 615, 631, and 661 nm followed by a µCT scan with 50 kV tube voltage and 0.24 mA tube current ensuring a minimal radiation exposure of 2 mGy per scan. BLT and µCT data were automatically reconstructed with MILabs BLT Recon software (MILabs) and 3D organ segmentation and quantification were performed based on the organ/tumor boundaries visible in µCT data, using Imalytics Preclinical software 2.1 (Gremse-IT) 46, 53. Segmentation of spleens was conducted using a sphere (r = 10 mm) due to insufficient soft tissue contrast. To compensate for the signal shift arising from deep tissue penetration of the bioluminescent light, spleen and lung segmentations were dilated by a factor of 10 voxels (Figure S1) 54. For comparison of the treatment groups, organ bioluminescence was normalized to the amount of total signal in the segmented mouse on each measurement day.

### *Ex vivo* analysis

#### Cyclic Immunofluorescence staining

Cyclic IF staining was performed as previously described 46, 54. Freshly excised xenograft tumors were embedded in Tissue Freezing Medium (Leica) and stored at -80 °C until further use. Sections of 8 μm thickness were cut on a CM3050 S cryostat (Leica) and collected on SuperFrost^®^ Plus slides (Menzel). Serial sections were either used directly or stored at -70 °C not longer than 2 weeks. For immunofluorescence staining slides were thawed in -20 °C acetone and staining of sections was performed with the fluorochrome-labeled antibody human CD3 (REA613, Miltenyi Biotec) together with 4',6-Diamidino-2-phenylindol (DAPI) (Sigma-Aldrich) according to manufacturer´s instructions. Finally, stained sections were covered with Fluorescence Mounting Medium (Dako) and coverslip. Images for ROI definition were acquired with EVOS^®^ FL Cell Imaging System (Thermo Fisher Scientific) and analyzed using ImageJ 1.49v.

Upon ROI definition, the subsequent tumor slice of the same frozen specimen was thawed in 4% paraformaldehyde (PFA) at room temperature. Residual PFA was removed 3 times with autoMACS™ Running Buffer (Miltenyi Biotec). Fixed sections were stained with DAPI (Miltenyi Biotec) according to manufacturer's instructions and stored at 4 °C until further usage. For cyclic IF staining, slides were introduced into the fully automated MACSima™ Imaging System, which conducts sequential stainings of multiple fluorochrome-labeled antibodies 46. Cyclic staining of the sections was performed with human-reactive antibodies against CD3, CD4, CD8, CD28, CD38, CD39, CD44, CD45RO, CD45RA, CD45RB, CD54, CD62L, CD99, CD152, CD154, CD161, CD183, CD223, CD278, CD279, CD326, EGFR, TIM3 as well as a mouse-reactive CD31 antibody (Miltenyi Biotec). Data processing and ROI stitching was conducted using MACS^®^ iQ View PPP (Miltenyi Biotec). MACS^®^ iQ View Analysis Software (Miltenyi Biotec) was applied for cell quantification using the following parameter: Advanced Morphology for Tissue, Nucleus Diameter: 18-60 px, Constrained Donut with CD44, CD3 and EGFR, Cytoplasm Sensitivity 100%, Cytoplasm Donut width 11 px, Nucleus Detection 90% and Nucleus Separation 70%. Cells in the vicinity of lints, out-of-focus area and outliers (5.7% of all cells outside the 3x standard deviation interval) were excluded from further analysis. For the remaining cells, MACS^®^ iQ's Biomarker Expression Feature was extracted, the cells were log2-transformed and the signal background mode was set to 0 for better comparability. A CD3 threshold was chosen heuristically for each treatment group to cover all CD3-bright areas and to discriminate between CD3-negative and -positive cell populations. Subsequently, the mean fluorescence intensity frequencies of the markers of interest were compared to each population and the cell types (CD4- and CD8-positive) frequencies within each treatment group sample.

### Immunostaining for 3D imaging analysis and xenograft clearing

Tumors for 3D image analysis were selected based on the average BLI signal and tumor size of the group and prepared for light-sheet fluorescence microscopy (LSFM) using the MACS^®^ Clearing Kit (Miltenyi Biotec). Vascular networks of tumors were stained *in vivo* with rhodamine lectin (Vector Laboratories). For this, 50 µL of rhodamine lectin (emission maximum 575 nm) were injected i.v. into the tail vein 5 min before mouse euthanasia. Excised tumors from the sacrificed mice were fixed by overnight incubation at 2-8 °C in 4% PFA Buffer. Remaining PFA was removed by washing in PBS 3× before tissues were permeabilized in 5 mL Permeabilization Solution (Miltenyi Biotec) per sample for 24 h at room temperature and under slow continuous rotation on a MACSmix™ Tube Rotator (Miltenyi Biotec). Subsequently, the tumors were stained with CD3-Vio667 (Miltenyi Biotec) in Antibody Staining Buffer (Miltenyi Biotec) for 7 days at 37 °C under gentle shaking. Unbound antibody was removed by washing 3× in Antibody Staining Buffer (Miltenyi Biotec) for at least 4 h each at RT under slow continuous rotation MACSmix™ Tube Rotator (Miltenyi Biotec). Stained tissues were dehydrated by incubation in increasing ethanol dilutions from 30% to 100% for at least 4h at RT under slow and continuous rotation. Following dehydration, tissue clearing was conducted using 5 mL MACS^®^ Clearing Solution (Miltenyi Biotec) under incubation for at least 6 h at RT under slow and continuous rotation.

### Image acquisition and 3D LSFM data processing

Cleared tumors were transferred into the imaging chamber of the light-sheet microscope (Ultramicroscope Blaze, Miltenyi Biotec) filled with MACS^®^ Imaging Solution (Miltenyi Biotec). Multichannel image acquisition of z-stack was performed dependent on sample size at a magnification of 1× or 4× with a zoom of either 0.6 or 1.66 or 4 µm step size. Background signal and stainings of rhodamine lectin and CD3-Vio667 were acquired in the following channels: 488, 561 and 640 nm, respectively. Subsequent analysis was performed with Imaris 9.5.1v and ImageJ 1.49v software (Bitplane). Specifically, 3D rendering of images and the determination of the tumor surface and volume, CD3-positive areas, and vessels were conducted. Soft tissue surrounding the tumor was separated by creating a mask based on manual delineation of the tumor margin. Voxels outside the mask were set to zero. Vasculature was segmented and reconstructed based on rhodamine lectin staining using the surface detection application. Analysis of CD3 stained areas was performed by surface segmentation or with Imaris “Spots” detection application. For spot detection, the cellular diameter was defined to not deceed 8 µm. CD3-positive areas were color-coded based on their relative location to the tumor surface or the cell density. The proximity of vessels to each other and different sizes of analyzed tumor determined the maximum distance. ImageJ 1.49v software was applied to investigate the gray values of the CD3 and rhodamine lectin staining at maximum projection within the middle third of the tumors.

### Statistics

Experimental results were analyzed using GraphPad Prism 9 software (Graph-Pad Software, USA). For the statistical comparison of two groups, unpaired t-tests were used. Analysis of two or more groups was conducted by one-way ANOVA with p < 0.05. Significance analyses of* in vivo* experiments were organized in a pairwise significance matrix (PSM). A comparison between two groups is represented by one box, as shown by Al Rawashdeh *et al.* 54. The order of group comparison is illustrated in Figure S2. Significant differences between two comparing groups are defined by a green box, while insignificant differences are indicated by a red box.

## Results

### Therapeutic efficacy of EGFR CAR T cells contrasts with 2D signal of the transgenic cells within the tumor

To investigate the pharmacokinetics of CAR T cells in relation to target recognition, EGFR CAR T cells and BDCA-2 CAR T cells were applied to EGFR-expressing tumor-bearing mice (Figure 1A). *In vivo* cell tracking was performed as outlined in Figure 1B. Two hours after CAR T cell injection, 2D BLI imaging displayed a signal in the lung as a result of the i.v. injection route (Figure S3) 55. On the next day, both treatment cohorts were further subdivided into two groups: for one group treatment was continued without further modification, while the other group additionally received IL-2 subcutaneously at the tumor site on day 1, 3, 5, and 7 post therapy initiation to assess the cytokine's impact to therapeutic efficacy. Subsequent longitudinal 2D BLI imaging demonstrated an accumulation of EGFR CAR T cells ± IL-2 at the tumor site in the following two weeks, while control-treated animals exhibited a signal in an area attributable to the spleen in dorsal and ventral view (Figure 1C). From day 13 on, bioluminescence became detectable in the spleen and other organs of EGFR CAR T cell-treated cohorts indicating that the T cells were either leaving the tumor site or amplifying in other organs to detectable levels. BLI scans of BDCA-2 CAR T cell-treated groups revealed strongly increasing signals over time in regions corresponding to the lower abdominal cavity and the head (Figure 1C). Continuously increasing bioluminescence in dorsal, ventral, and tumor ROIs were quantified for further analysis with similar sized ROIs for all groups and timepoints (Figure 1D). The comparison of dorsal signal development - without tumor area - showed no significant difference between EGFR CAR T cell and EGFR CAR T cell + IL-2 treated animals. For BDCA-2 CAR T cell + IL-2 treated animals, a significantly higher signal was observed in dorsal and ventral ROIs, which persisted even after the discontinuation of additional IL-2. However, we were not able to detect any differences between the tumor signals of the four treatment groups. But these results contrasted the clear intensity peak at the tumor sites of EGFR CAR T cell treated animals visible on day 6 and day 13 (Figure 1C), and the significant tumor reduction in response to the EGFR CAR T cells at the endpoint of the analysis assessed by 2D caliper measurement (Figure 1E) 56.

### 3D hybrid µCT/BLT visualizes antigen recognition-dependent intratumoral CAR T cell trafficking and expansion over time

**Figure 2:**
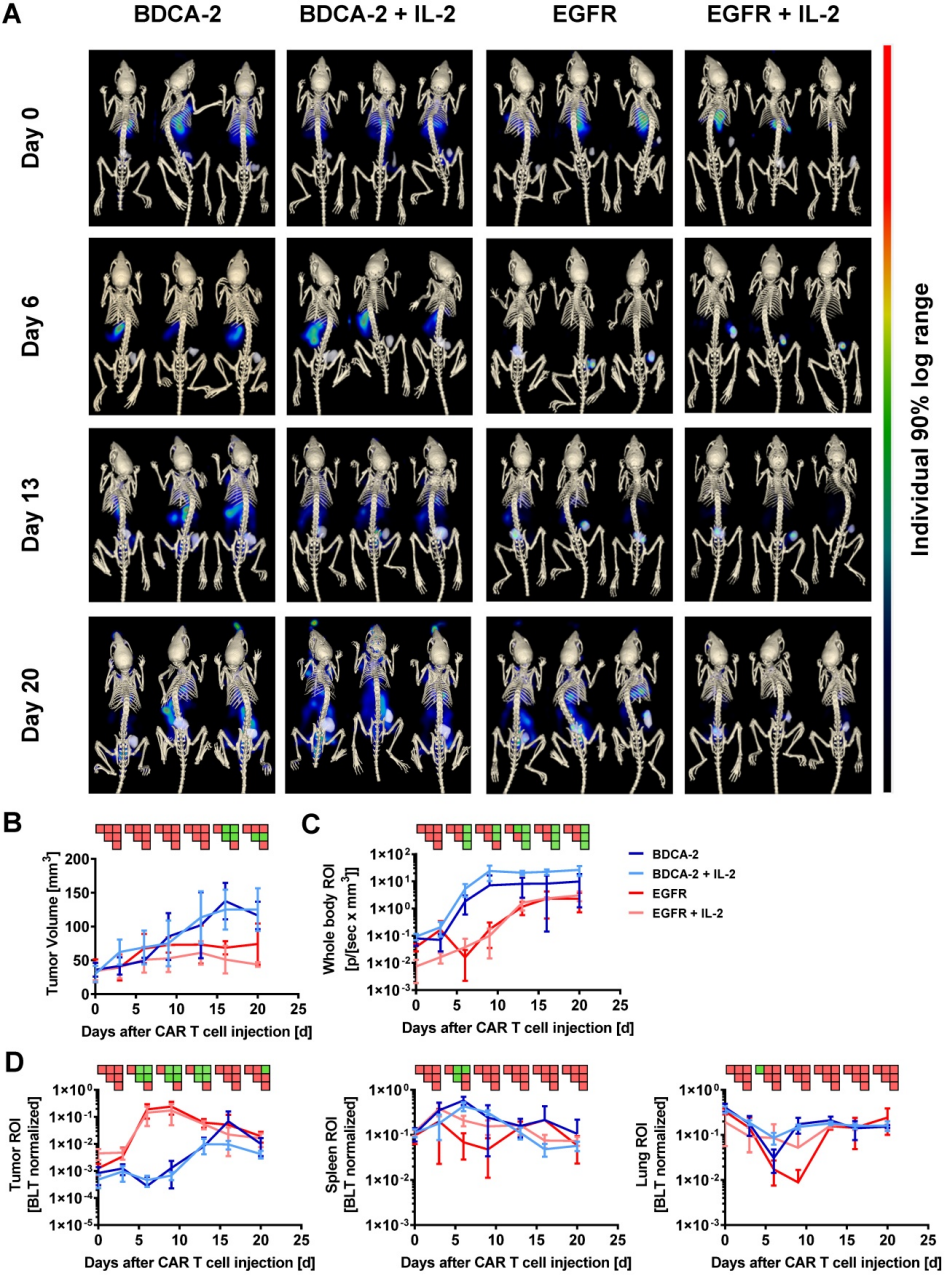
**3D hybrid µCT/BLT precisely detects antigen recognition-dependent intratumoral CAR T cell trafficking and expansion**. EGFR CAR T cells were clearly visible in the tumor and 3D hybrid µCT/BLT quantified significantly higher EGFR CAR T cells numbers than the control treatments on 3 separate measurement days. **(A)** Representative *in vivo* 3D BLT scans of CBR2opt-transduced CAR T cell distribution in NSG mice. **(B)** Tumor burden change over time measured by µCT scans. Quantification of bioluminescence amplification within the entire animal body **(C),** and at tumor site and in the segmented spleen and lung **(D).** PSM p < 0.05 (green).

Subsequent to the 2D BLI measurements, 3D µCT/BLT measurements of *in vivo* CAR T cell distribution were performed. The majority of the animals within all four groups displayed a signal peak within the rib cage, originating from the lungs at the baseline measurement on day 0 (Figure 2A). On day 6, EGFR CAR T cell treated animals displayed a clear influx of CBR2opt expressing CAR T cells into the tumor at the right flank of the mouse. In control groups, BLT imaging revealed a dominating signal in the spleen accompanied by a lack of signal in the tumor, in contrast to 2D BLI imaging. On day 13, BLT measurement showed that EGFR CAR T cell treated animals appeared to have two distinct signal peaks matching with tumor and spleen site. However, the background signal started to increase after this time point. The same was true for BDCA-2 CAR T cell treated groups, which further showed a diluting spleen signal in favor of amplifying signals e.g. in the lower abdominal cavity. Additionally, tumor influx of BDCA-2 CAR T cells in AsPC1 BDCA-2^-^ negative tumor started to manifest on day 13. At the endpoint of the experiment, EGFR CAR T cell influx was not the dominating signal anymore due to enhanced signal in the whole mouse caused by the development of xenogeneic graft-versus-host disease (GvHD). Here, visual examination of the BLT scans did not result in the detection of any differences in the CAR T cell biodistribution in the presence or absence of subcutaneous IL-2 injection under the tumor. Consequently, volume determination of the tumor size by μCT revealed a significant therapeutic effect by EGFR CAR T cells in contrast to BDCA-2 CAR T cell treated animals, which was slightly increased by additional IL-2 between day 6 and day 13, however without any statistical significance between EGFR CAR T cell and EGFR CAR T cell + IL-2 CAR T cell treated groups (Figure 2B). The more objective µCT based tumor volume assessment showed no significant anti-tumor efficacy in contrast to the highly user-sensitive 2D Caliper measurement on certain time-points (Figure 2B). The overall CAR T cell distribution was also quantified, by organ segmentation and signal quantification of the segmented organs (Figure 2D). Longitudinal BLT measurements of all four groups demonstrated an increase of bioluminescence over time in the whole mouse, indicating CAR T cell proliferation, but the slope varied strongly dependent on the CAR and the presence of additional IL-2 (Figure 2C). Mice treated with EGFR CAR T cells experienced a strong signal decrease between day 0 and day 3, which was prevented by local IL-2 at day 1. Recovered signal of EGFR CAR T cell and intensity of EGFR CAR T cell + IL-2 treated animals reached a plateau at day 13, indicating a steady-state phase of T cell proliferation and death. Signal in BDCA-2 CAR T cell and BDCA-2 CAR T cell + IL-2 treated animals increased continuously from day 0 on without a strong signal decline at the beginning, nor resulting in any anti-tumor effect. In contrast to 2D BLI analysis of the tumor ROI, 3D BLT quantification of the segmented tumor enabled a clear quantification of the antigen-specific CAR T cell proliferation and accumulation at the tumor site, by normalization to the signal increase over time in the whole segmented mouse (Figure 2D). This strong CAR T cell expansion came to an end after day 13, aligning with a plateau of the anti-tumor effect in EGFR CAR T cell treated animals between day 16 and day 20, indicating an end of the active tumor-killing phase. During the same time, BDCA-2 CAR T cell treated groups demonstrated only a slowly increasing signal between day 0 and day 20. Nevertheless, by BLT we could not identify an additional effect of IL-2 on EGFR CAR T cell and BDCA-2 CAR T cell expansion at the tumor, since an overall higher signal in the whole mouse ROI was detected in IL-2-treated mice. Subjective visual analysis of the signal peak in the spleen suggested a T cell specific infiltration in all groups. However, quantification of the bioluminescence within the spleen ROI could not corroborate the visual impression (Figure 2D). Taken together, we demonstrated significant target-dependent trafficking of CAR T cells to the tumor correlating with tumor efficacy by applying 3D BLT imaging.

### Spatial analysis of tumor-infiltrating T cells using LSFM reveals increased presence of T cells in the tumor periphery

**Figure 3:**
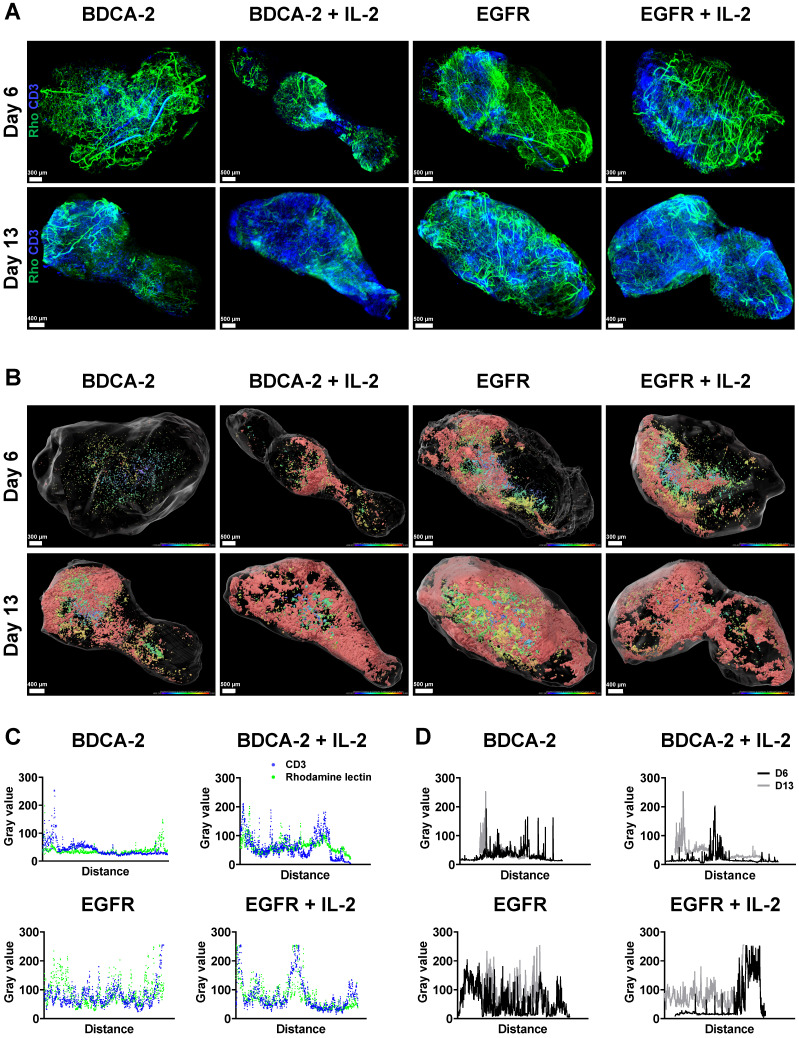
** LSFM indicates a target-dependent advantage for CAR T cell distribution at an early time-point.** Reconstruction and color coding of CD3-positive cells intratumoral distribution indicated a spatially heterogeneous distribution and deeper tumor penetration in the EGFR CAR T cell-treated groups. **(A)** 3D rendering of excised tumors (n = 1/group/day) with T cells (*ex vivo* CD3-Vio667, in blue) and vasculature (*in vivo* rhodamine lectin, in green) staining. **(B)** Projection of reconstructed CD3-positive cells distribution using a color gradient reflecting T cells distance from the tumor surface, Red: closest, Blue: furthest. **(C)** Colocalization of maximum intensity projection gray value profiles of CD3-positive cells and vasculature on day 13 across each tumor. **(D)** Overlay of maximal intensity projected gray values of CD3-positive cells on day 6 and 13 across each tumor.

**Figure 4:**
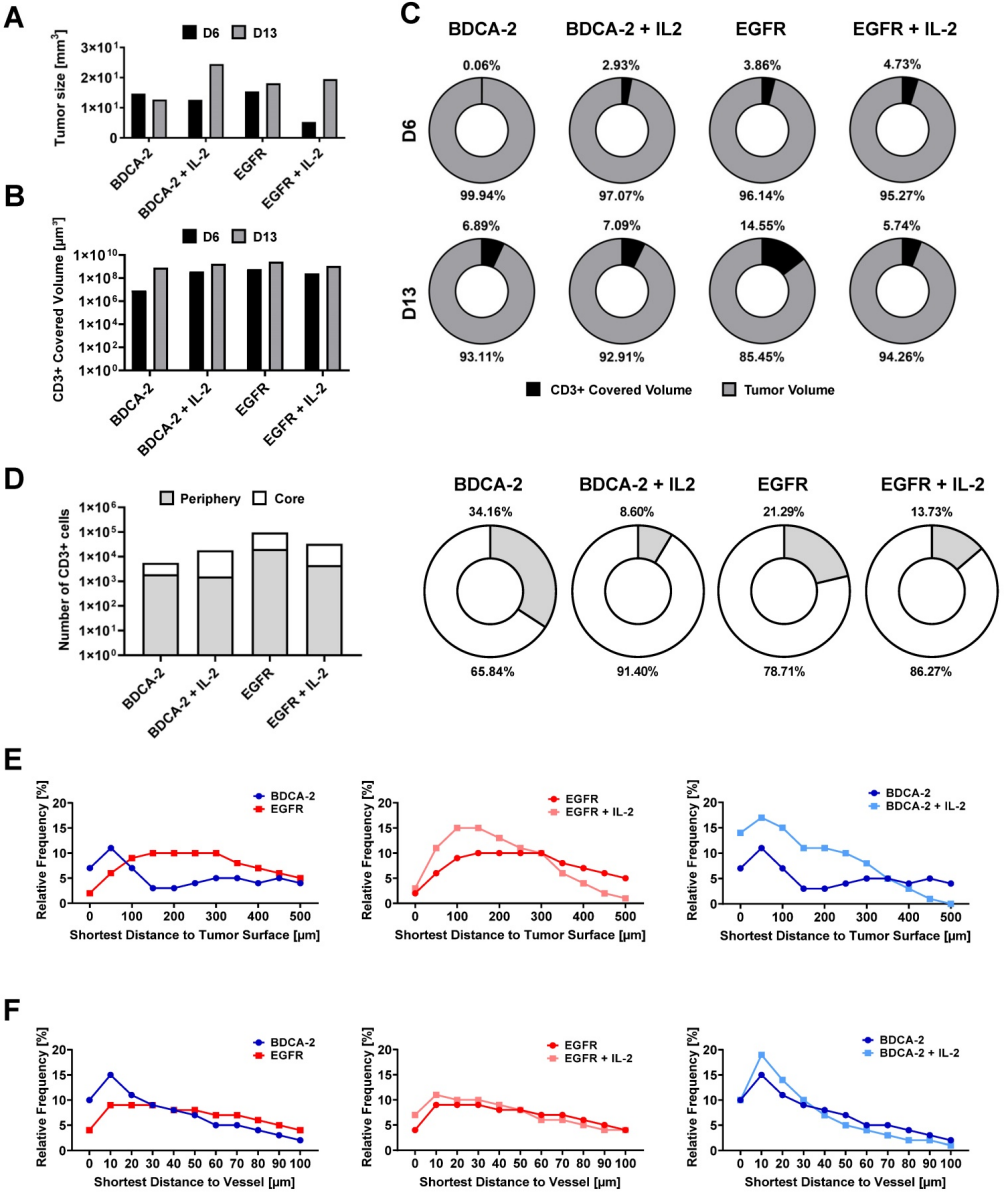
** Spot-reconstruction analysis in LSFM reveals effects of IL-2 on CAR T cells intratumoral distribution.** Subcutaneous IL-2 application near the tumor induces target unspecific CAR T cell proliferation in the tumor periphery but does not contribute to enhanced tumor penetration nor enhanced vasculature extravasation. **(A)** Tumor sizes used for LSFM analysis on day 6 and day 13. **(B)** Volume of surface reconstructed CD3-positive areas within tumors. **(C)** CD3-positive volume percent. **(D)** Percentage of CD3-positive cells in the tumor periphery compared to in the tumor core on day 6. Equal amounts of reconstructed CD3-positive spots were assigned to each region. **(E)** Relative frequency of tumor-infiltrating spot-reconstructed CD3-positive cells in the tumor, with 0 representing the tumor surface. **(F)** Distance of spot-reconstructed CD3-positive cells to the vasculature, with 0 representing a volume-reconstructed vessel surface (n = 1/group/day).

To better understand the interaction of the therapeutic cells with the tumor as well as the impact of additional IL-2 supply, 3D LSFM imaging at single-cell resolution was conducted on tumors excised on week 1 and 2. Visualization in 2D revealed the presence of different levels of CD3-positive cells depending on the applied treatment. Strikingly, a significant amount of T cells was located in close proximity to large feeding vessels, which represent important sources for T cell transport and nutrient supply 57 (Figure 3A). Subsequent 3D reconstruction of the tumors in combination with distance color-coding for CD3-positive cells displayed a spatially heterogeneous, island-like distribution of the intratumoral T cells that resided primarily in the outer tumor layers (Figure 3B). Notably, during week 1, tumors of EGFR CAR T cell-treated mice exhibited a higher T cell infiltration rate than those of BDCA-2 CAR T cell-treated mice (Figure 1C, 2A, 3B, S4). Intensity profile analysis excluded the possibility of an inefficient antibody penetration where it revealed the lack of a gradient staining (Figure S5). In both cohorts the addition of IL-2 was associated with only a minor increase of intratumoral CD3-positive cells, as indicated by ~3% increase in the BDCA-2 CAR T cell- and ~1% increase in the EGFR CAR T cell treated tumors on day 6, respectively (Figure 3B, 4C).

Intratumoral CD3-positive cell distribution was further quantified via the gray values of the CD3 staining at the maximum projection in the middle third of each tumor from both weeks (Figure S6) and showed that the majority of T cells were located close to the tumor surface and vasculature (Figure 3C, 3D). Gray value comparison further revealed an increased T cell accumulation at the tumor margin (Figure 3D). The EGFR CAR T cell-treated tumors had the highest T cell infiltration with a negligible difference between week 1 and 2, while BDCA-2 CAR T cell treated tumor revealed the lowest T cell infiltration, regardless of the time point (Figure 3D). IL-2 increased the overall profile intensity in the target-specific and unspecific groups (Figure 3D). In-depth analysis of the intratumoral immune cells using the IMARIS spot reconstruction for CD3-positive cells and segmentation of the tumor core and periphery confirmed that only a small fraction was able to reach the core (Figure 4D). Spot detection further enabled distance measurements of the reconstructed CD3-positve spots to the vasculature and the tumor surface (Figure 4E, 4F). Target specificity increased the amount of CD3-positive in close distance to the tumor surface (Figure 4E). However, from a distance of 400-500 µm inwards, BDCA-2 and EGFR CAR T cells exhibited a comparable infiltration frequency (Figure 4E). Additional IL-2 supply resulted in a higher frequency of CD3-positive cells within the outer 300 µm of the tumor margin, independent of the target specificity. The relative frequency of CD3-positive cells to the vessel network in a certain distance was also assessed (Figure 4F). Strikingly, only a minimal impact of IL-2 could be detected on the penetration capacity of T cells. Rather, the infiltration appeared to be guided by target recognition.

To delineate the most active sites of T cell proliferation, intratumoral T cells were then clustered based on their density (Figure S7). T cells with the highest density were found to locate primarily at the outer layers of the tumors, emphasizing that the proliferative capacity of the immune cells decreases with further penetration into the tumor. Moreover, EGFR CAR T cell-treated tumors displayed the highest T cell density within these tissues indicating that there was the strongest anti-tumor and proliferative response ongoing. Surprisingly, however, an IL-2-mediated stimulus on T cell proliferation was observed in LFSM only for the control treatment in analyzed tumors in the second week. For the EGFR CAR T cell-treated cohort, T cells appeared to benefit only short-term from the pro-inflammatory cytokine supply on day 6 (Figure 4C).

### Local administration of IL-2 at the tumor site enhances T cell proliferation and activation, resulting in increased exhaustion

**Figure 5:**
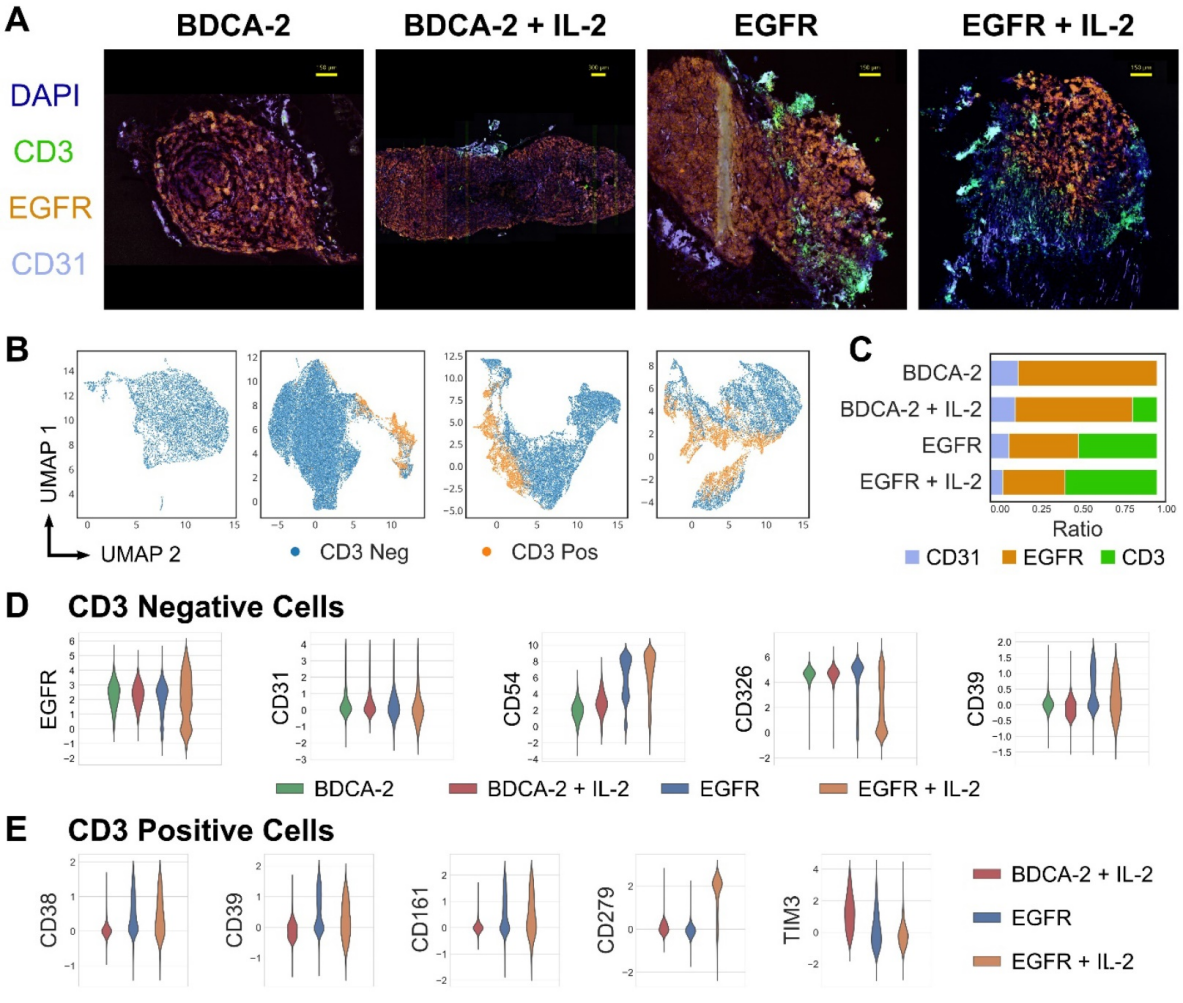
** High-content cyclic IF imaging reveals phenotypic changes in tumor and T cells following CAR T cell treatment.** A library of antibodies was screened to characterize the tumor cell:T cell interactions and to deep phenotype intratumoral T cells using the MACSima™ Imaging Platform. **A)** Composite images of EGFR, CD31, CD3 and DAPI staining from tumor samples of each treatment group. Following multi-field imaging, ROIs were stitched to yield a full overview of the tumor sections. **B)** UMAP projection of cellular components in tumor samples from each treatment group and the discrimination of CD3-positive and CD3-negative cells. **C)** Relative frequency of CD31-, EGFR-, and CD3-positive cells per treatment. **D)** Distribution of EGFR, CD31, CD54, CD326, and CD39 expression in the CD3-negative compartment per treatment type. **E)** Distribution of CD38, CD279, CD161, CD39 and TIM3 expression in the CD3-positive compartment in the EGFR CAR T cell treated groups with or without IL-2 addition and the BDCA-2 CAR T cell + IL-2 treated tumor.

Having assessed the intratumoral distribution of T cells in 3D, multi-dimensional IF analysis was performed to deep-phenotype the infiltrating T cells. Consistent with previous observations, tumors treated with EGFR CAR T cells±IL-2 displayed a higher degree of T cell infiltration than the cognate control groups (Figure 5A, C). Moreover, the EGFR signal intensity levels of malignant tissue were lowered in the presence of EGFR CAR T cells but not BDCA-2 CAR T cells, indicating that the engineered therapeutic cells actively attacked the antigen-expressing tumor cells (Figure 5A). Uniform Manifold Approximation and Projection (UMAP)-based analysis of CD3-positive and -negative cells displayed differing amounts of tumor and T cell clusters, suggesting phenotypic differences in both cell types depending on the nature of therapy (Figure 5B). An in-depth analysis of the tumor composition displayed a compliant expression pattern between EGFR and the epithelial marker CD326, whose levels decreased upon EGFR CAR T cell+IL-2 treatment indicating the highest tumor cell death in this cohort. By contrast, treatment with EGFR CAR T cells led to an upregulation of the adhesion molecule CD54 on tumor cells, with further augmentation upon IL-2 administration. Similarly, CD39 - a protein involved in the inhibition of T cell proliferation - was upregulated upon EGFR CAR T cell therapy, but not following BDCA-2 CAR T cell administration (Figure 5D). Specifically on T cells, an upregulation of the checkpoint molecules PD-1 (CD279), CD161 and CD39 was observed upon EGFR targeting. In particular PD-1 was significantly upregulated upon IL-2 administration of EGFR CAR treated tumors. Taken together, CAR T cell therapy in solid tumors induced phenotypic changes in tumors and activated T cells and the effects were further augmented upon IL-2 administration (Figure 5E).

## Discussion

Despite three decades of intensive efforts, CAR T cell therapies for solid cancers have not yet been approved for clinical use. This lack of success is partially due to an inability in the current preclinical setting to thoroughly characterize novel CAR T cell therapies, so that most severe adverse events are being identified in the clinical testing and lead to a cessation of the respective therapeutic development. With the now increasing awareness that CAR T cell applications are associated with toxicities, immune suppression, low CAR T cell tumor infiltration and exhaustion, the need is growing for methodologies to properly evaluate CAR T cell therapies prior to medical translation. Ideally, these methodologies should enable longitudinal analyses as - owing to their nature as living drugs - CAR T cells actively traffic throughout the body and undergo dynamic expansion and contraction kinetics which cannot be comprehended by end-point analyses. Thus, current efforts focus on imaging-based approaches applying BLI and/or PET to study CAR T cell characteristics on a whole-body scale with temporal reconstruction 26-28, 30-32, 34, 35. In this context, Sellmyer and colleagues proved the combination of BLI with PET particularly advantageous as it harnesses the high detection sensitivity of BLI with an improved spatial resolution of PET/CT 27. While a step in the right direction, the limitations of this approach are the use of radionuclides, the need for two reporters and a small throughput of image acquisitions. To overcome these drawbacks, in this study, we applied µCT/BLT for the analysis of CAR T cell biodistribution. Using the recently described bioluminescence reporter CBR2opt, which was shown to convey superior sensitivity in deep tissue imaging 48, as low as 5∙10^5^ transgenic cells could be tracked *in vivo*. Compared to 2D BLI, cognate BLT data facilitated the quantitative and anatomical analysis of the bioluminescent source and thus allowed improved pharmacokinetic evaluation of CAR T cells. Consistent with previous reports, we observed initial lung sequestration of systemically administered CAR T cells 34, 35 followed by a reduced overall number of CAR T cells entering the circulation as indicated by a whole-body bioluminescent intensity drop between day 0 and 5. Thereafter, antigen-activated and -nonactivated CAR T cells displayed distinct accumulation kinetics: while in the EGFR CAR T cell (± IL-2)-treated cohorts, the first signal emerged at the tumor site, BDCA-2 CAR T cells (± IL-2) primarily homed to the spleen. Although this observation is in line with several other studies 34, 58, 59, Sellmyer and colleagues reported discrepant findings in which both therapeutic and control T cells initially homed to the spleen followed by an accumulation at the tumor site 27. This contradiction can be explained by the study of Skovgard and colleagues who demonstrated that CAR T cell accumulation occurs in an antigen-dependent manner resulting in tumors with high antigen load exhibiting early CAR T cell accumulation whereas in antigen low lesions the immune cell accumulation proceeds relatively slow 34. In fact, in our model, EGFR is an abundantly expressed tumor antigen (Figure S8), whilst Sellmyer and colleagues used GD2 for targeting which is expressed heterogeneously and at relatively low levels in tumor cells 60.

An intriguing observation in our study was that, initially, EGFR CAR T cells displayed a pronounced proliferation at the tumor site followed by a decay of the bioluminescent signal indicating a reduction of intratumoral CAR T cells in the second half of the therapy. One possible explanation for the limited CAR T cell persistence may be the high antigen load in the tumor and the strong binding activity of the Cetuximab-scFv, which lead to an onset of activation-induced cell death (AICD) in the CAR T cells 61-63. In light of the continuous tumor control, however, it is also plausible to assume that the T cell numbers decrease due to tumor volume reduction resulting in reduced tumor retention. This observation has been described in previous work and the hypothesis is further supported by a growing CAR T cell signal in the spleen starting the onset of therapeutic efficacy in the anti-EGFR-treated cohorts 32.

One practical challenge in *in vivo* cell tracking is the choice of suitable controls. Non-activated T cells show limited persistence which is accompanied by rapid signal loss, in particular, when low cell numbers are administered. To overcome this obstacle, we used the control CAR for BDCA-2 that was tested to provide low-level tonic signaling in this way augmenting the T cell persistence. However, contrary to our expectations, we observed a stark signal increase throughout therapy indicating that additional mechanisms were in play. It is likely that in addition to tonic signaling donor T cells became activated against recipient-specific antigens and elicited graft-versus-host diseases (GvHD). Endpoint *ex vivo* organ imaging in both groups confirmed strong bioluminescence signals in xenogeneic GvHD associated organs, including intestine, lung and ovaries 64. In contrast to EGFR CAR T cells which are actively eradicating tumor cells, BDCA-2 CAR T cells are more potent in their GvHD response.

To our knowledge, this is the first study using µCT/BLT for the characterization of cell therapies *in vivo*. The necessity of one reporter gene only in combination with the 3D information that can be acquired in high throughput and under cost-effective conditions provides a platform for accelerated and improved preclinical development of CAR T cell therapies. Nevertheless, two limitations should be taken into consideration when applying bioluminescent imaging: 1) The hypoxic conditions within solid tumors can reduce the availability of ATP and O_2_ which may lead to diminished bioluminescent signal of intratumoral CAR T cells 65. 2) Current BLT technology is restricted in single cell tracking abilities, therefore limiting the information on the spatial organization of the transgenic cells on a sub-organ level 44, 66. To overcome these obstacles, we introduced high-resolution LSFM into our workflow, for which tumors were taken out on selected therapy points and imaged for T cell localization in 3D. We observed that in all treatment groups the trafficking of the immune cells into the tumor core was compromised and the majority of T cells accumulated in the tumor periphery, which is consistent with clinical observation and reported to be counteractive for efficient anti-tumor activity 67-69. One predominant reason for the spatially restricted distribution of T cells in pancreatic cancers is known to be desmoplasia which results in increased deposition of extracellular matrix components around the lesion in this way creating a physical barrier to T cells. However, early clinical results seen with T cell vaccines suggest that T cells nevertheless have the capability to infiltrate the PDAC microenvironment 70. Thus, in an attempt to increase the therapeutic index, we tested CAR T cell therapy with additional IL-2 supply. Although the T cells indeed displayed an increased proliferative capacity, this effect was restricted to the tumor surface. Strikingly, the overall penetration depth decreased for both treatment groups upon IL-2 administration. It is likely, that the metabolic requirements associated with T cell activation were not met in the highly immunosuppressive TME so that the effector function was mainly restricted to the tumor surface which is rich in nutrients and O_2_ supply. Thus, CARs providing a strong activating signal as it is known for Cetuximab-based CARs, appear not to benefit from additional IL-2 supply in cancers with a suppressive TME.

Besides spatial organization, studies suggest that the phenotype of the therapeutic cells is a decisive factor for therapeutic efficacy as well. Specifically, the expression of the inhibitory molecules PD-1, TIM-3 and LAG3 was reported to identify malfunctioning T cells in different tumors. In our study, we observed that CAR T cell therapy against PDAC has an effect on both the tumor and the therapeutic cell phenotype. Upon EGFR CAR T cell treatment, the tumor cells displayed an upregulation of the adhesion molecule CD54 that was unseen in control-treated tumors. Moreover, a concomitant upregulation of CD39 was identified, a molecule that is known to promote tumor cell growth and suppress immune cell responses 71. While the underlying mechanism of these observations needs to be elucidated, it is tempting to speculate that this represents an escape mechanism, by which the tumors attempt to evade the immune cells: Upon upregulation of CD54, whose binding partner LFA is expressed on T cells, the tumor cells immobilize T cells after which secondary inhibitory molecules such as CD39 suppress T cell function.

Besides tumor cells, expression of CD39 was also observed on therapeutic T cells, which was previously reported to function as a checkpoint inhibitor on the immune cells 38. Intriguingly, previous work reported the co-expression of CD39 and PD-1 to identify functionally exhausted T cells 72, 73. This is significant as in our study, we observed that additional IL-2 supply at the tumor site led to a drastic upregulation of PD-1 expression and induced a tendency towards an exhaustive T cell state. Thus, while modifications of the TME towards a pro-inflammatory state may represent a promising approach to improve T cell efficacy, these approaches also warrant caution as they may result in opposite effects.

## Conclusion

Overall, our study revealed for the first time that non-invasive imaging and longitudinal monitoring of CAR T cell distribution *in vivo* is feasible using a hybrid 3DµCT/BLT imaging system. Moreover, 3D CAR T cell tracking demonstrated the correlation of increased antigen-specific CAR T cells at the tumor with therapeutic efficacy. LSFM and cyclic IF proved to be advantageous for the analysis of CAR T cell-tumor cell interactions, assessment of CAR T intratumoral distribution at cellular resolution and analysis of IL-2 influence on CAR T cells, which altered CAR T cell proliferation, location, and phenotype within the tumor.

## Supplementary Material

Supplementary figures.

## Abbreviations

AICD: activation-induced cell death
BBB: blood-brain-barrier
BLI: bioluminescence imaging
BDCA-2: blood dendritic cell antigen 2
CAR: chimeric antigen receptor
CBR2opt: click beetle red luciferase 2 optimized
CRS: Cytokine release syndrome
EGFR: Epidermal Growth Factor Receptor
FMT: fluorescence molecular tomography
GvHD: Graft-versus-Host-Disease
IF: Immunofluorescence
IL-2: Interleukin-2
i.p.: intraperitoneal
i.v.: intravenous
LSFM: light-sheet fluorescence microscopy
NSG: NOD scid gamma
OI: optical imaging
PET: Positron emission tomography
ROI: region of interest
RT: room temperature
s.c.: subcutaneous
SPECT: single photon emission computed tomography
TME: tumor microenvironment
UMAP: Uniform Manifold Approximation and Projection
µCT/BLT: µComputer tomography bioluminescence tomography
2D: 2-dimensional
3D: 3-dimensional
