# Supplementary material for: A multimodal imaging workflow for monitoring CAR T cell therapy against solid tumor from whole-body to single-cell level
Source: Theranostics. 2022 Jun 13;12(11):4834–50. doi: 10.7150/thno.68966 (PMC9274742; doi:10.7150/thno.68966)
Supplement: Supplementary file 1 — Supplementary figures. [file thnov12p4834s1.pdf]

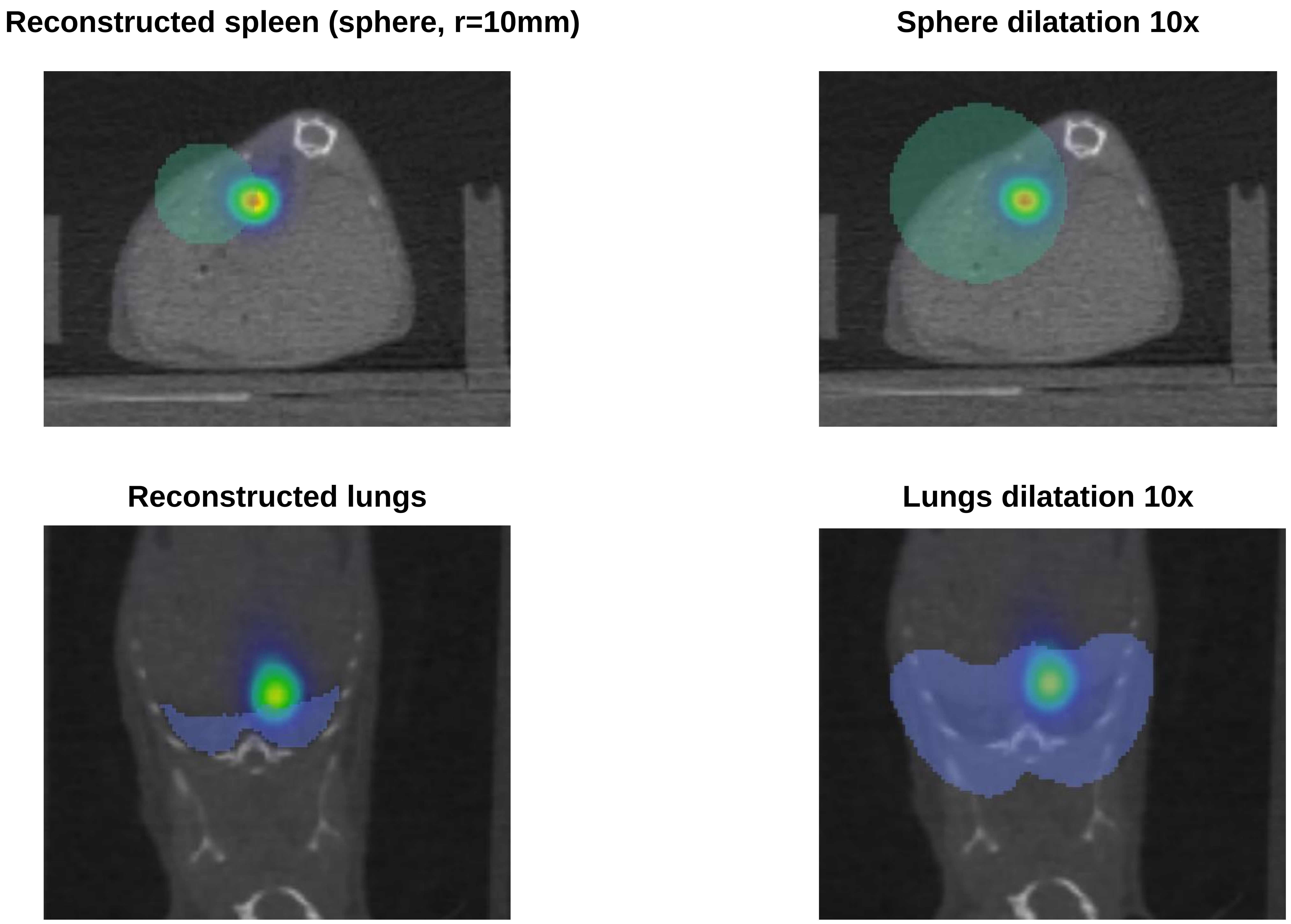

Figure S1: Compensation of BLT signal shift in spleen and lungs. View of representative spleen (upper) and lungs (lower) signal on Day 0 and Day 3 in BDCA-2 treated mice. Spleen was reconstructed using a sphere (r = 10mm) (green) based on CT scan. Lungs were reconstructed using contrast-based thresholding. Shifted signal was included into the redspective spleen and lungs ROI by dilatation of the ROI by factor of 10 in x-, y- and z dimension.

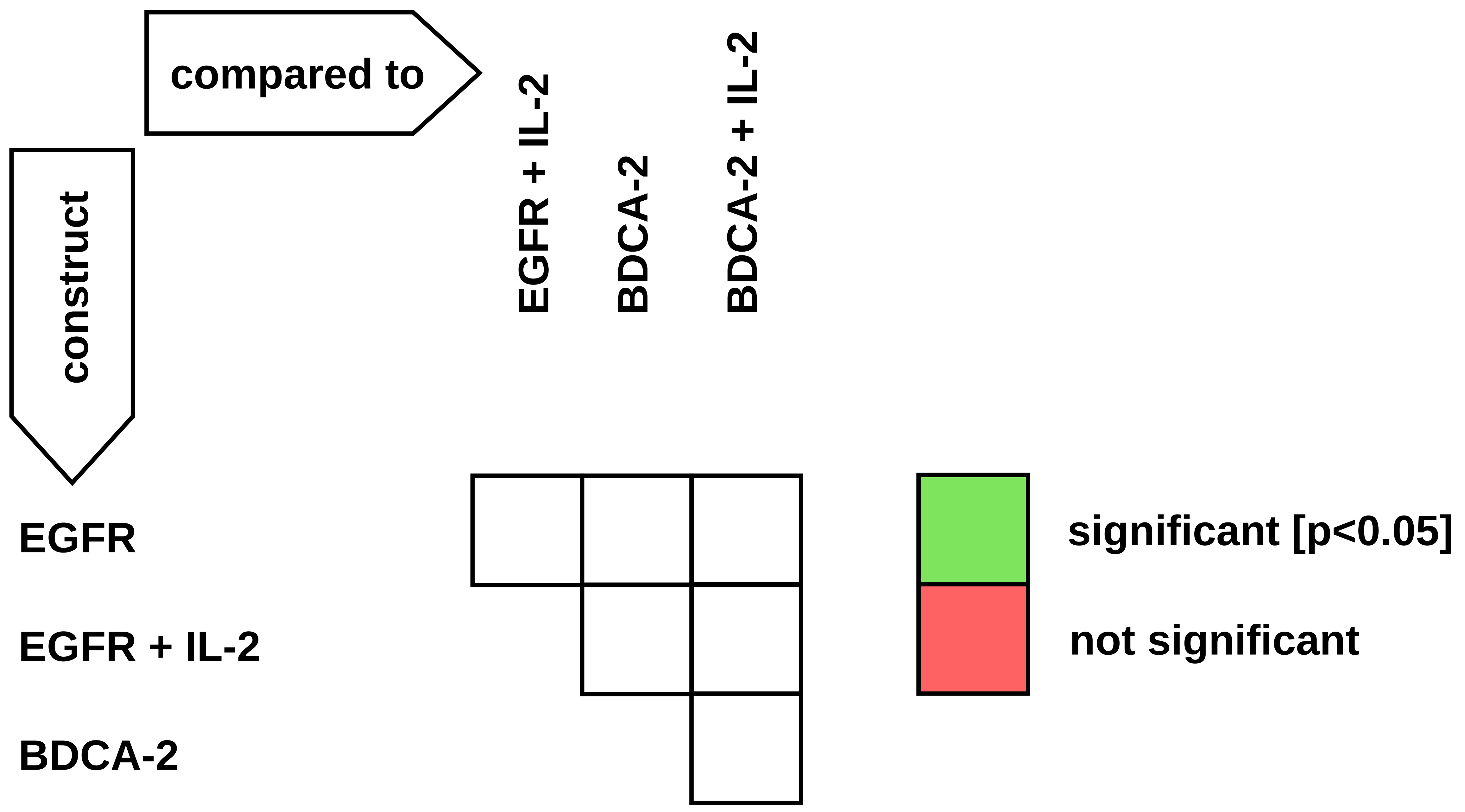

Figure S2: Organization of the pairwise significance matrix for group comparisons. BLT and BLI of EGFR, EGFR + IL-2, BDCA-2 and BDCA-2 + IL-2 CAR T cell treated tumors were quantified and compared. PSM $p < 0.05$ (green), $p > 0.05$ (red) [one-way ANOVA, multiple comparisons].

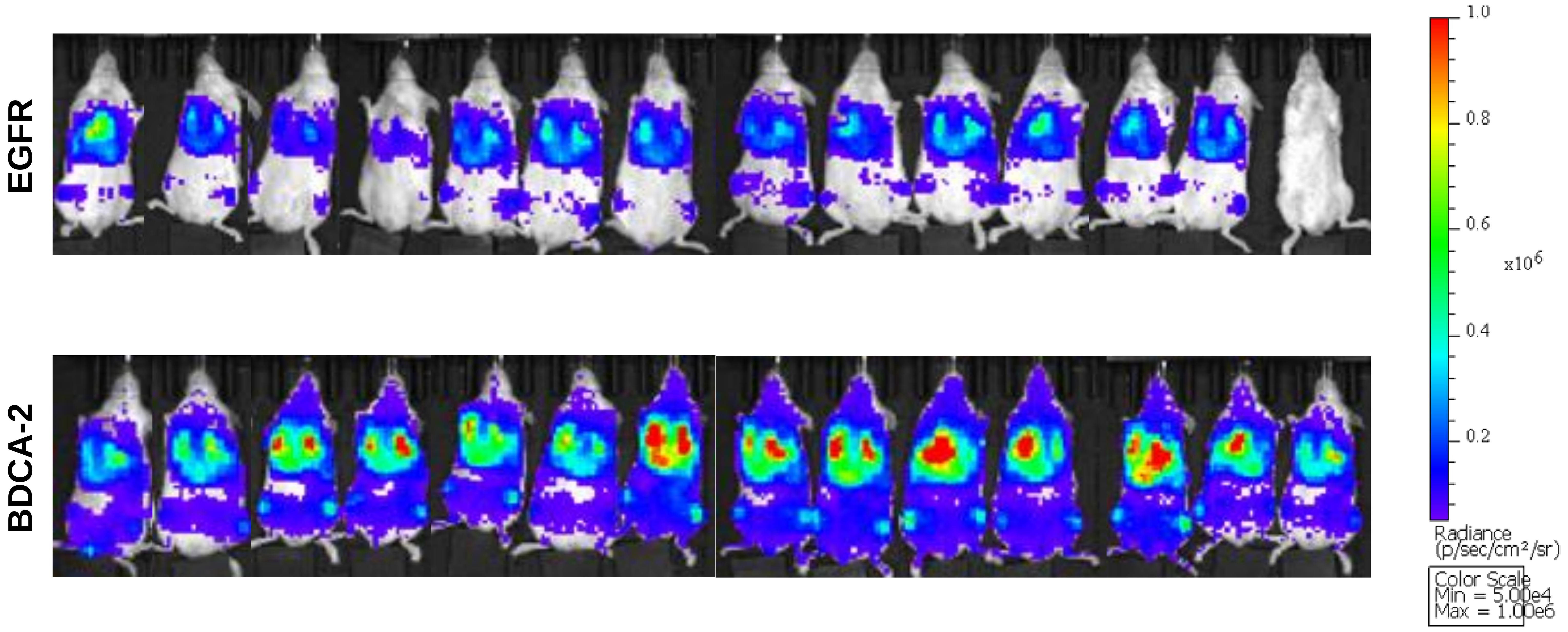

Figure S3: Homogenous distribution of CAR T cells, 2h after injection on Day 0. CAR T cells were injected intravenously via the tail vain. 2h post-injection, mice were intraperitoneally injected with 100 µl D-Luciferin and measured at the IVIS Lumina *in vivo* imaging system.

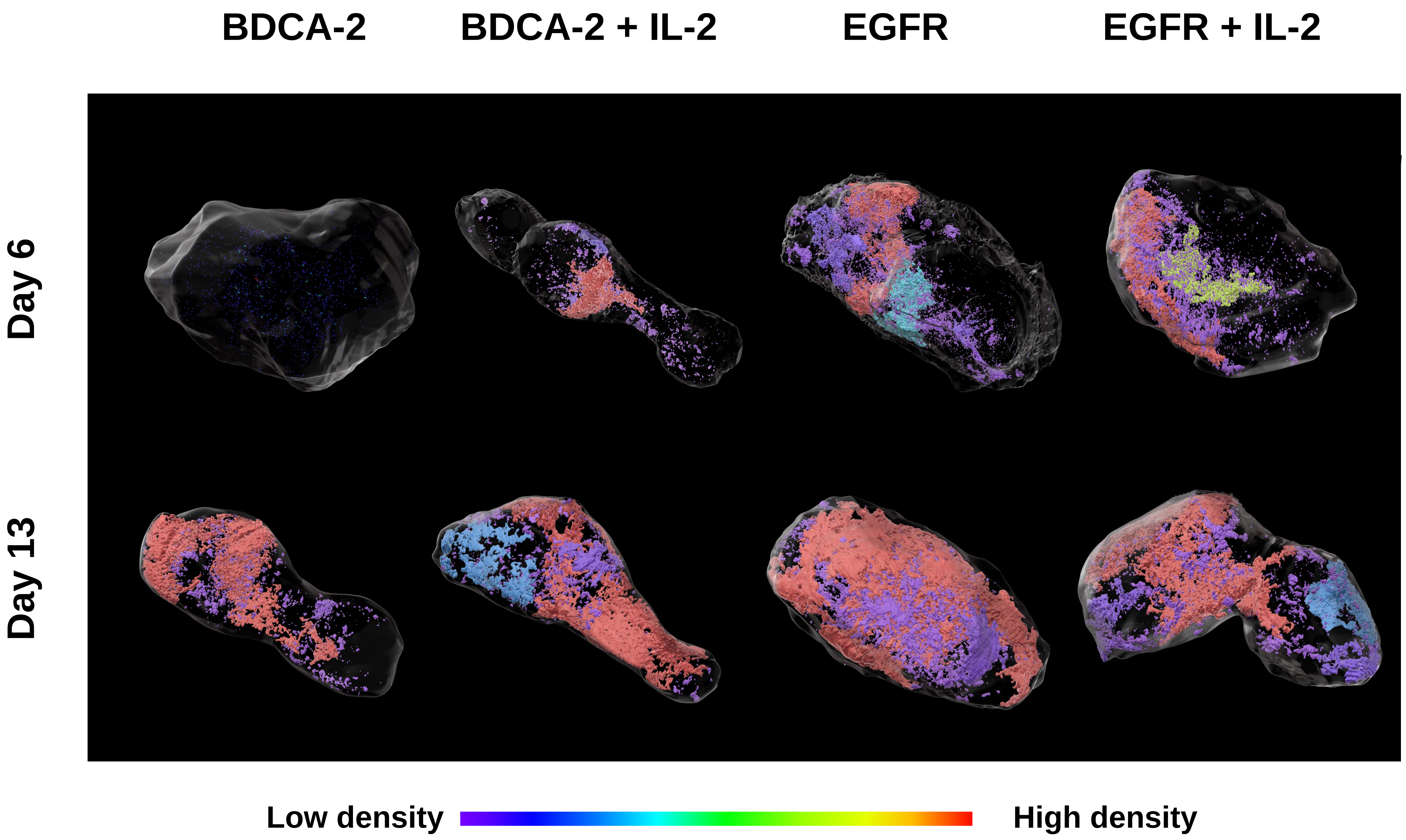

Figure S4: Density Clustering of intratumoral T cells on Day 6. Imaris reconstruction was applied and CD3-positive areas were color-coded based on staining intensity, in order to identify areas in the tumors with the highest density of T cells.

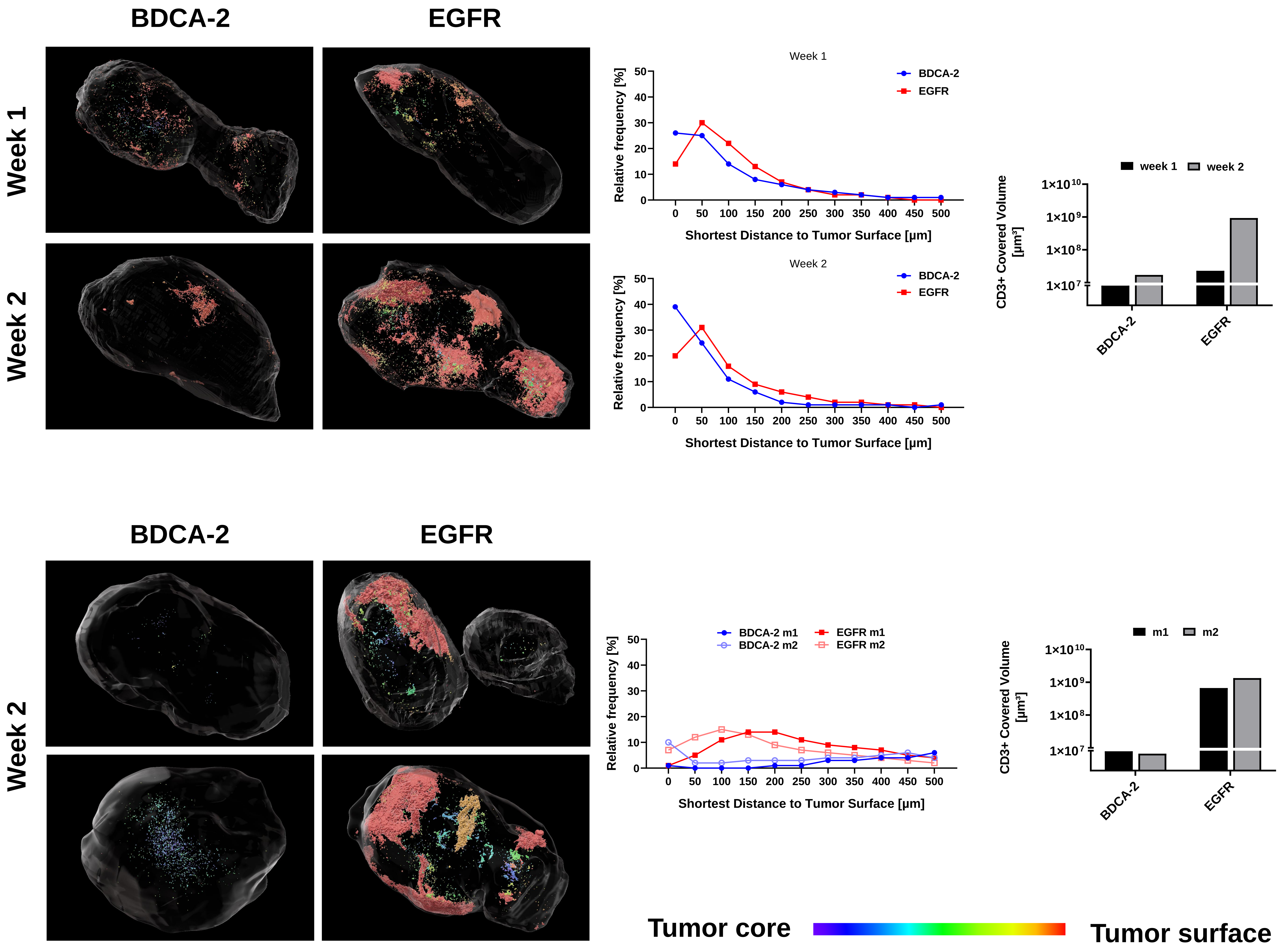

Figure S5: Analysis of CAR T cell distribution in solid tumors on single cell level. Spatial information about the localization of transgenic T cells was collected in various, independent *in vivo* studies using *ex vivo* 3D LSFM. CAR T cell treated tumor samples were excised in the first or/and second study week. Post-processing of LSFM scans was done using Imaris Bitplane, where CD3-positive areas were segmented and color-coded based on their distance to the tumor surface. Relative frequencies of CD3-postive cells were displayed over the distance and CD3+ covered tumor volumes were quantified for each sample.

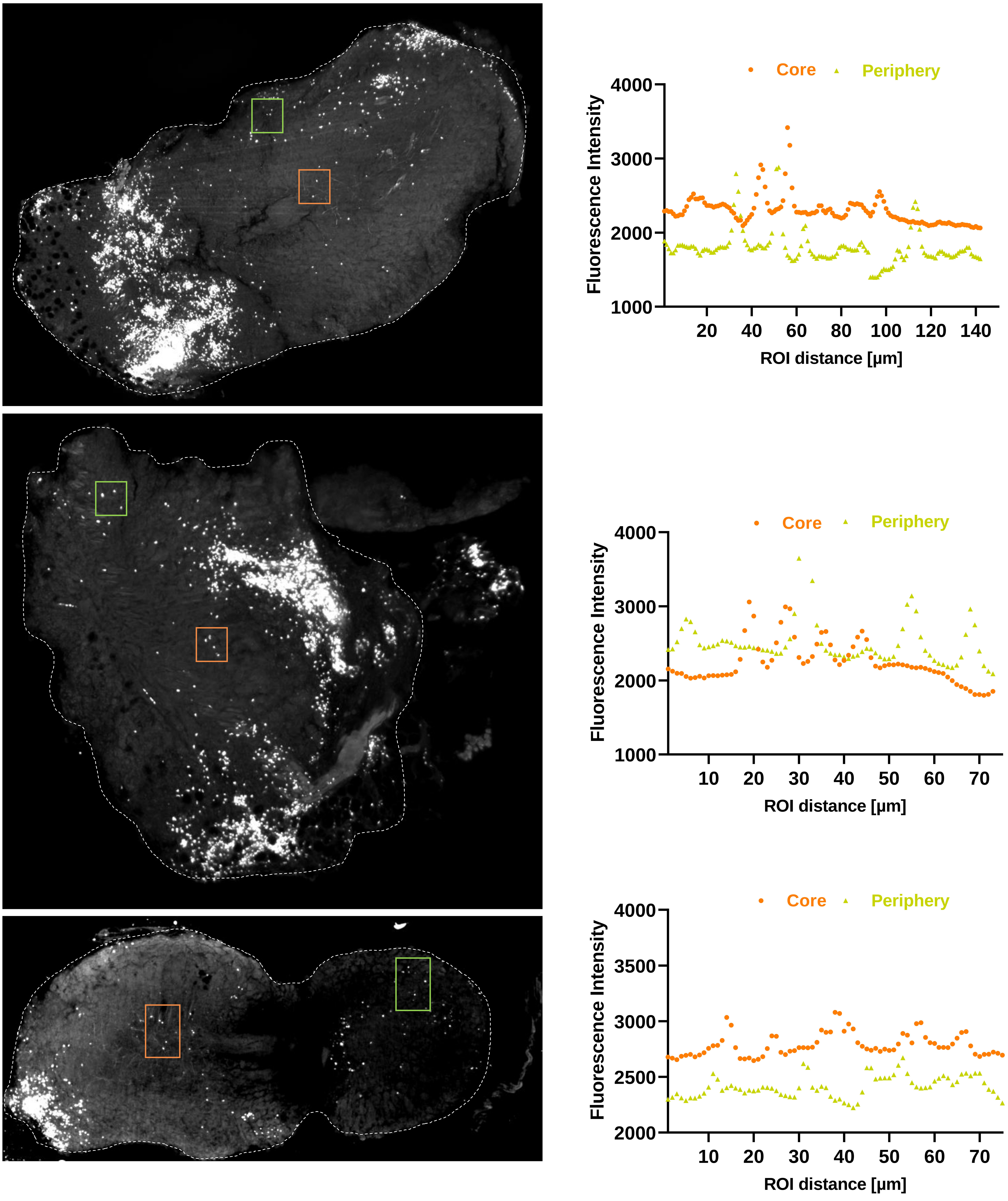

Figure S6: Fluorescence intensity profile analysis. In order to evaluate antibody penetration, CD3 staining intensity was analyzed in each sample using the central 20-30 µm of the tumor, respectively. For this, the central 5 z-sections were displayed in a maximum intensity projection, rectangular ROIs were selected in the tumor core and periphery covering only individual cells and gray values were analyzed using Fiji ImageJ.

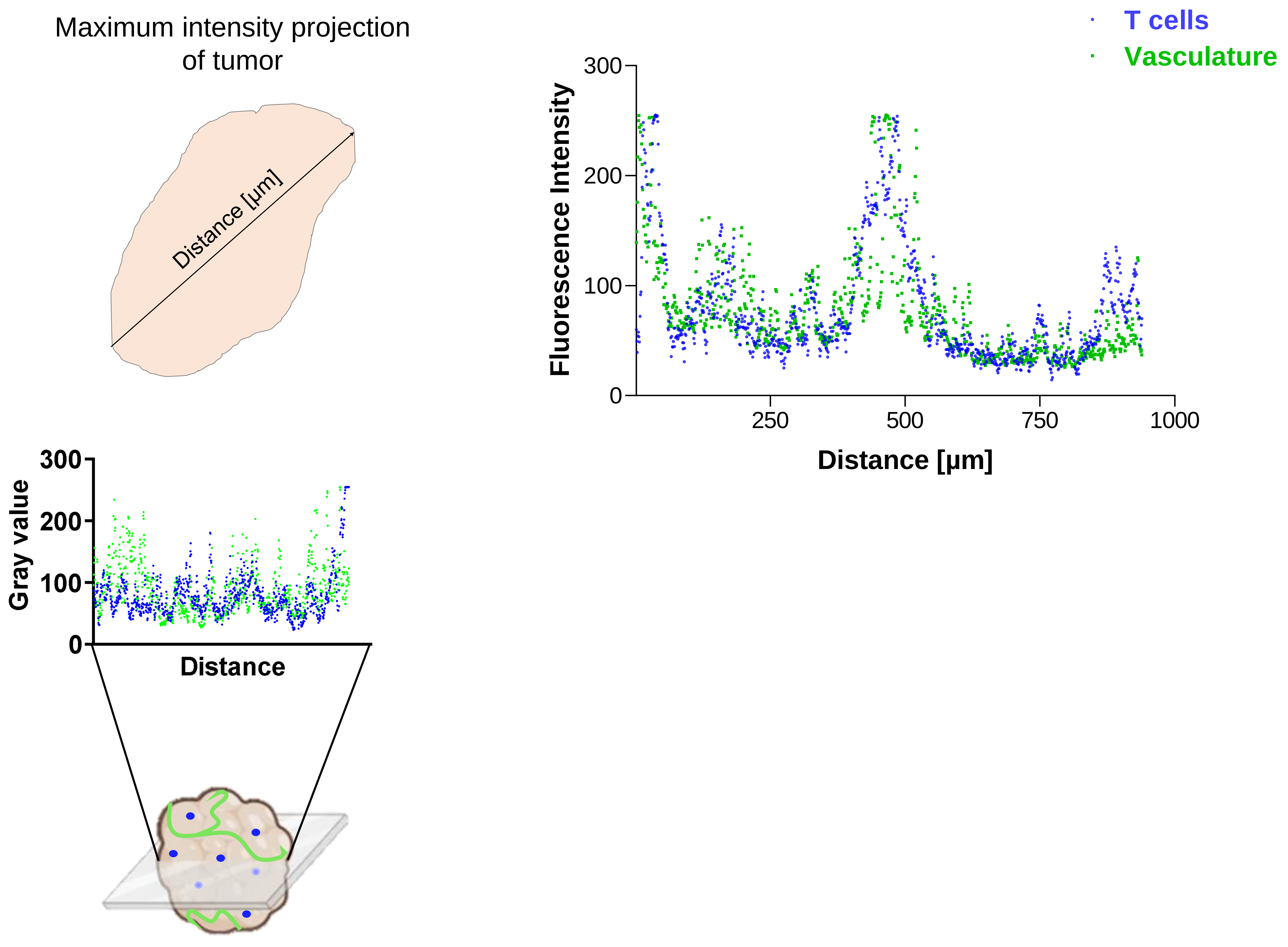

Figure S7: Depiction of maximum intensity projection gray value profiles analysis. The middle third of the tumor was selected for each tumor respectively and either maximum intensity of the vasculature (green) or of the CD3-positive stained areas (blue) were detected. An overlay of both maximum intensity profiles was generated to analyze co-localization of vessels and T cells.

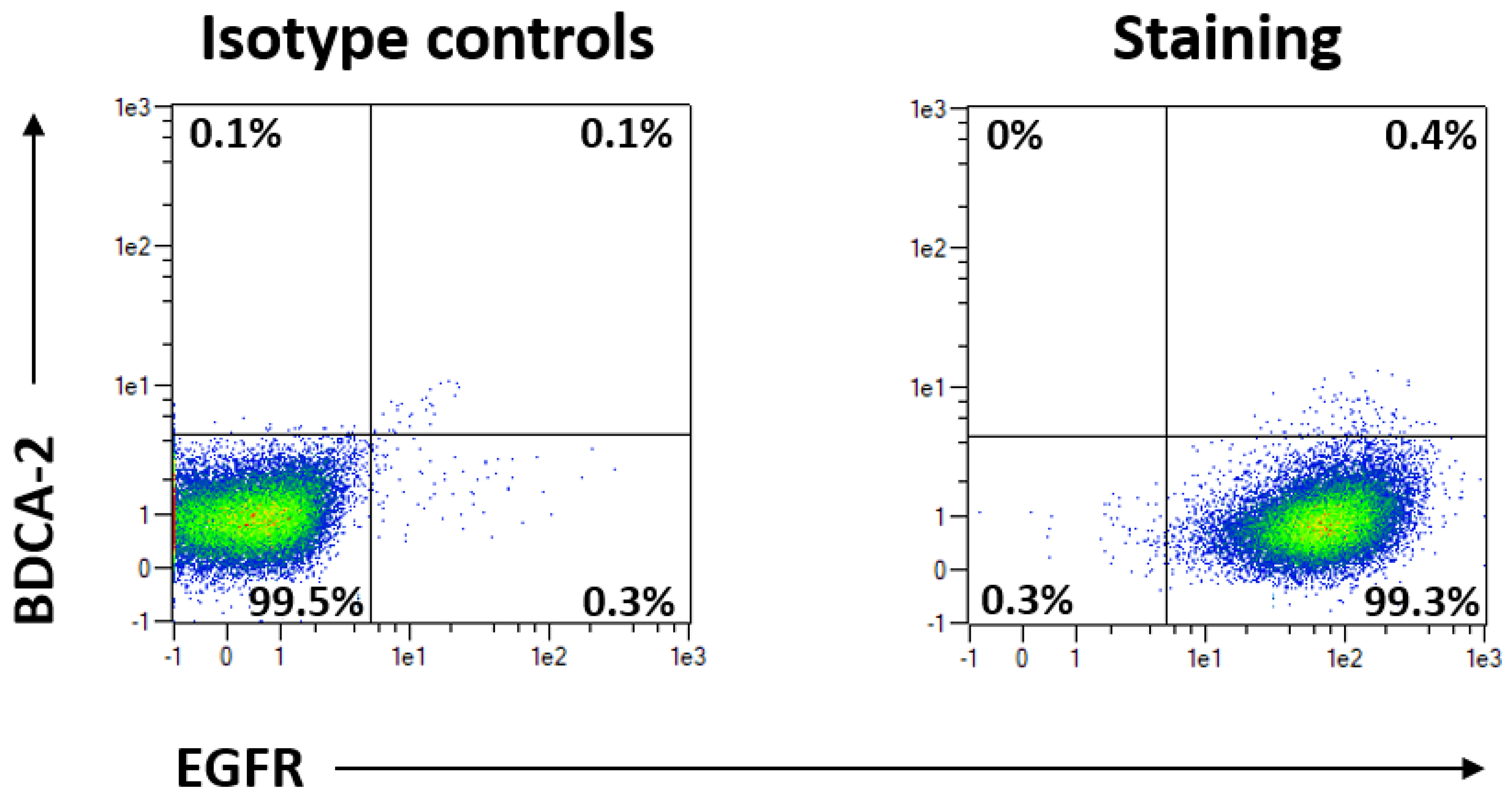

Figure S8: Surface expression of BDCA-2 and EGFR on the pancreatic cancer cell line AsPC-1. Antigen expression was determined by flow cytometry using the BDCA-2-specific antibody AC114 and EGFR-directed antibody REA939.
